# Substance use prevention services in juvenile justice and behavioral health: results from a national survey

**DOI:** 10.1186/s40352-020-00114-6

**Published:** 2020-05-13

**Authors:** Rodney Funk, Hannah K. Knudsen, Larkin S. McReynolds, John P. Bartkowski, Katherine S. Elkington, Ellen H. Steele, Jessica M. Sales, Christy K. Scott

**Affiliations:** 1 Chestnut Health Systems, 448 Wylie Drive, Normal, IL 61761 USA; 2 College of Medicine, Department of Behavioral Science and Center on Drug and Alcohol Research, University of Kentucky, 845 Angliana Avenue, Room 204, Lexington, KY 40508 USA; 3 Vagelos College of Physicians and Surgeons, Department of Psychiatry/New York State Psychiatric Institute, Columbia University, 1051 Riverside Drive, Unit 78, New York, NY 10032 USA; 4 Department of Sociology, University of Texas at San Antonio, One UTSA Circle, San Antonio, TX 78249-0655 USA; 5 Department of Psychiatry, Columbia University and New York State Psychiatric Institute, 1051 Riverside Drive, #15, New York, NY 10032 USA; 6 Social Science Research Center, Mississippi State University, 1 Research Boulevard, Suite 103, Mississippi State, MS 39762 USA; 7 Rollins School of Public Health, Department of Behavioral Sciences and Health Education, Emory University, 1518 Clifton Road NE, Room 570, Atlanta, GA 30322 USA; 8 Chestnut Health Systems, 221 West Walton Street, Chicago, IL 60610 USA

**Keywords:** National survey, Substance use prevention, Juvenile justice, Community supervision, Juvenile probation, Community behavioral health providers

## Abstract

**Background:**

This study examined the national availability of substance use prevention (SUP) within juvenile justice (JJ) and their primary behavioral health (BH) providers, and the relationships between the availability of SUP and agency-level measures of organizational structure, staffing, and youth characteristics. A three-stage national probability sampling process was used to select participants for a national survey that included, among other facets of community supervision (CS) and BH practices, questions on agency characteristics, youth characteristics, whether the agency/provider directly provided SUP services, and whether the agency/provider directly provided substance use and/or mental health treatment. This paper focuses on SUP services along with agency/provider and youth characteristics related to providing SUP.

**Results:**

The response rate for both CS agencies (*n* = 195) and BH providers (*n* = 271) was 96%. Complex samples logistic regression initially examined univariate associations of each variable and identified candidates for a final multivariate model. Overall, only one-third of CS and BH providers reported offering SUP services, with BH providers being significantly more likely than CS agencies to provide SUP services. In addition, likelihood of SUP was significantly lower among agencies where the substance use distribution of the caseload was below the median. Controlling for master’s level staff and the substance use distribution, CS agencies were about 67% less likely to offer SUP when compared to BH providers.

**Conclusions:**

Given the high rates of substance use among justice-involved youth and that substance use is an established risk for several negative behaviors, outcomes, and health conditions, these findings suggest that evidence-based prevention services should likely be expanded in justice settings, and perhaps included as part of CS programs, even when youth do not initially present with SU service needs.

## Background

Substance use is common among justice-involved youth. Approximately 70% of arrested juveniles have had prior drug involvement (Belenko & Logan, 2003), over half of those entering community supervision currently have a substance use problem (Dennis et al., 2019; Scott, Dennis, Grella, Funk, & Lurigio, 2019), and over one-third have substance use disorders (Aarons, Brown, Hough, Garland, & Wood, 2001; Wasserman, McReynolds, Schwalbe, Keating, & Jones, 2010). Substance use has been closely associated with several negative behaviors and outcomes (e.g., delinquency, recidivism, school truancy and dropout, social and family dysfunction), as well as health conditions (e.g., psychopathology, risky sexual behaviors, and sexually transmitted infections) among this vulnerable population of adolescents (Hicks, Iacono, & McGue, 2010). Justice-involved youth tend to initiate substance use earlier than other adolescents, leading to more problematic substance use and continued involvement with justice authorities (Henggeler, Clingempeel, Brondino, & Pickrel, 2002; Hoeve, McReynolds, & Wasserman, 2013; Hoeve, McReynolds, Wasserman, & McMillan, 2013; Kandel & Yamaguchi, 2002).

If substance use, especially problematic use, is undetected and untreated among justice-involved youths, they are particularly likely to recidivate (Hoeve, McReynolds, Wasserman, & McMillan, 2013; Schubert, Mulvey, & Glasheen, 2011; White, 2019) and to continue offending into adulthood (Hoeve, McReynolds, & Wasserman, 2013). Thus, substance use prevention, both primary and secondary prevention efforts, for justice-involved youth is critically important to disrupt pathways to multiple potential adverse physical, mental and behavioral health outcomes, as well as to reduce the risk of recidivism. Substance use prevention programs aim to delay or prevent onset of use among those who have never used illegal substances (i.e., primary prevention), as well as to reduce escalation in use among those who have already begun using or relapse after abstinence (i.e., secondary prevention). Fortunately, many evidence-based substance use prevention programs exist. A review of evidence-based interventions for preventing substance use and abuse among youth highlights numerous exemplary prevention programs that can be implemented at the individual, group, family, school or community level (Griffin & Botvin, 2010; Knight et al., 2019; Shelton, 2005; Tanner-Smith, Durlak, & Marx, 2018).

Although prevention and treatment programs have been developed, tested and reviewed (e.g., https://www.crimesolutions.gov/), they remain inconsistently utilized for meeting the needs of justice-involved youth. National data on access to behavioral health services for justice-involved youth are lacking, yet prior studies have found that only 21–25% of youth referred for treatment services actually received services (Burney Nissen, Butts, Merrigan, & Kraft, 2006; Dennis et al., 2019). Moreover, having a diagnosis of a disorder is not predictive of receiving services (Sales et al., 2018). Although the juvenile justice system is well poised to deliver evidence-based treatments and prevention programming, such programs tend to be underutilized in justice settings (Leukefeld et al., 2017; Scott et al., 2019; US Census Bureau, 2010). For example, in a study of community supervision agencies in seven states, only 22% of agencies offered an evidence-based prevention program (Bartkowski, Xu, Avery, Ferguson, & Johnson, 2018).

Behavioral health services, particularly substance use treatment, are typically provided outside of the juvenile justice system through cross-systems partnerships with local behavioral health providers (Saunders et al., 2016). In a previous paper, only about one in ten CS agencies reported directly providing substance use and/or mental health treatment, while about 90% of BH providers directly provide these services (Scott et al., 2019). However, over a third of these agencies/providers were low on collaboration between these two systems. Positive change on this front is also thwarted by organizational and system dynamics, including external behavioral health providers’ reliance on diagnoses and treatment that are reimbursable by health insurance (Burney Nissen et al., 2006; Sales et al., 2018). Resources to support substance use prevention may be more difficult for behavioral health providers to acquire given that health insurance does not reimburse such services. At the same time, it is plausible that behavioral health organizations would be more likely to offer substance use prevention than juvenile justice agencies as the former have expertise and experience in direct service provision.

The absence of available information about the availability of prevention programming for justice-involved youth, in either the behavioral health system or the justice system, limits our ability to develop approaches to increase access to and uptake of these programs. Thus, as a critical first step, this paper sought to (1) describe the national availability of substance use prevention within juvenile justice community supervision agencies and their primary behavioral health providers, and (2) examine the relationships between the availability of substance use prevention and agency-level measures of organizational structure, staffing, and youth characteristics.

## Methods

### National Survey Description

This study uses empirical data collected from two organizational sources across the United States: (1) community supervision (CS) agencies in the juvenile justice system (JJS), and (2) their primary behavioral health (BH) providers of substance use and mental health treatment. These agencies play a vital role in processing adolescent offenders while aiming to address the complex constellation of needs often presented by this population. A survey instrument was generated to gauge various facets of CS protocol and practice evident in the JJS and the BH sector. The JJS CS surveys and the BH surveys included questions on data availability; agency characteristics; youth characteristics; behavioral health (substance use, HIV, and mental health) screening, clinical assessment and referral; substance use and HIV/STI-risk prevention; substance use and mental health treatment; and interagency collaborative activities, family engagement, and technical assistance needs. The questions also focused on whether services were provided directly or through referrals; the names and utilization of evidence-based tools, protocols, and other practices; staff educational levels; and training or experience needs.

### Sampling methodology

Respondent selection was based on a three-stage national probability sampling process that included states, counties, and CS agencies within counties. States and counties were stratified by the number of youth aged 10 to 19 residing in them, as documented in the 2010 Current Population Survey (Gottfredson et al., 2018). In the first stage, the five largest states were selected with certainty. The remaining 15 were selected with probabilities proportionate to the number of youth in five population strata to ensure that less populated states were included in the study.

Within each state, the largest county and any other mega-counties (those with 250,000 or more youth, or half or more of the state’s youth in smaller states) were selected with certainty. The remaining counties were selected with probabilities proportionate to the number of youth in those counties. In the two small sampled states organized by judicial district instead of counties, we included all counties/districts.

In the third stage, all CS agencies that served youth on CS in the 192 sampled counties were identified and surveyed (*n* = 203), regardless of the number of youth they served. In states where CS agencies were managed at the state level, key stakeholders at the state level were contacted to help identify the CS agencies and appropriate contact within the sampled counties and to encourage participation in the survey. In states with decentralized systems, all CS agencies within the sampled counties were contacted directly.

Within each sampled county, the selected CS agencies were asked to identify the primary behavioral health providers of substance use and mental health treatment (i.e., those that served the most youth under CS from their sampled county). In over two-thirds of the selected agencies, substance use treatment and mental health treatment were provided by the same agency, but the rest were provided by two different agencies. Most BH providers were external to the CS agency, but for a small number of CS agencies (*n* = 12), the BH provider was housed in an internal unit within their own agency. A total of 283 BH providers were identified in the 192 sampled counties.

### National survey administration, completion, and weighting

The agencies/providers were instructed to have staff that were familiar with the agency’s organization, priorities, youth under community supervision and the services they receive to fill out the survey. Instructions also stated that it was likely that several staff may need to provide input. Along with the survey instructions, each agency/provider had a survey coach to help increase the likelihood of accurate and complete responding. Regardless of how many helped put together the answer, each community supervision agency/primary healthcare provider was asked to submit one combined response and have the senior person from the agency sign the informed consent to participate in the national survey. All of the research design, implementation and analysis was conducted under the supervision of Chestnut Health System’s Internal Review Board for the protection for human subjects.

Surveys were completed by 195 of the 203 identified CS agencies, for a 96% CS organizational response rate. Data were weighted based on the inverse of the inclusion probability at each of the three stages (1.0 for certainty state or counties and stage 3) and then adjusted for non-response within state. The number of agencies overall and those providing a specific service were estimated by multiplying the weighted average number of agencies per county times the actual number of counties (*n* = 3143) in the United States (excluding the District of Columbia and territories). This calculation generated a national estimate of 3202 CS agencies serving youth under CS.

Of the 283 BH providers identified, 271 surveys (96%) were completed and returned, again yielding a 96% response rate. The total number of BH service providers overall was estimated based on the weighted average number of BH service providers per county times the number of counties (*n =* 3143 counties). The number of BH providers providing each specific service were estimated by multiplying the weighted average number of providers providing the service times the number of BH service providers (*n* = 4252). The data for this analysis combines the records from both the CS survey and the BH survey. This strategy yielded a weighted total of sample of 7454 agencies/providers (unweighted total is 480).

### Measures

In this study, the primary outcome variable of interest is whether the respondent from the CS agency or BH provider reported directly providing substance use prevention (SUP) services to clients under community supervision. The survey included a list of 57 evidence-based prevention programs; the list also included non-evidence based programs such as DARE. Respondents were asked to endorse any of these or if they used a locally developed program or some other program that was not on the list. Directly providing SUP was defined as the respondent endorsing one or more prevention programs from this list. For more on the survey and SUP items, see Scott et al. (2019). The evidence-based program list was based on the peer-reviewed programs enumerated in the federal Crime Solutions website (CrimeSolutions.gov, n.d) that were rated as having a promising base or strong base of evidence.

To control for county size, we dichotomized counties into “rural” (Beale codes 4, 5, 6, 7, 8, 9) coded as “1” or Urban area counties (Beale codes 1, 2, 3) coded as “0”. We retrieved these codes from https://www.ers.usda.gov/data-products/rural-urban-continuum-codes/.

Other agency-level variables, including agency type, staffing, and caseload characteristics, were included as independent variables. Of primary interest was agency type, with CS agencies coded as “1” and BH providers coded as “0.” For staffing, we used the number of full-time equivalent (FTE) doctoral level employees and the number of master level FTEs.

Caseload characteristics included agency/provider-level measures of demographics and substance use characteristics of youth on CS served by the agency in the past year. Respondents were asked to report the percentage of youth on CS served during the past year who were female and the percentage who were racial and ethnic minorities. Respondents could also report if the data for each item was either not accessible or not collected by the agency/provider. About 23% of the agencies/providers surveyed had missing data on female clients, 23% had missing data on minorities, and 21% were missing data on age. Given the high rates of unknown or missing responses on the percentage of female youth caseload, we coded each answer into one of 3 groups: (1) unknown/missing, (2) below the median (less than 30%), or (3) at or above the median (30% or more). For percentage of caseload that was from a minority group, we summed the percentages reported across African American, Native American, Asian, Hispanic, multi-racial, or other races and maxed at 100%. The minority caseload median spit was < 8% and > =8%. The percentages of youth in the age ranges 14–15, 16–17 and age 18+ were summed and so the age 14+ caseload median was based on < 86% and > =86%.

Respondents were asked to report the percentages of the youth caseload with alcohol problems, marijuana problems, and prescription drug problems. Since these three variables are correlated, we constructed a summary variable across these three substances by using the maximum value across these three variables to divide agencies/providers into one of three groups. If all percentages were unknown, we coded the agency/provider into the substance use unknown group. If the respondent reported percentages for the three substances that were never above the median, the agency/provider was coded as being below the median, and if one or more values were above the median, we coded the agency/provider as being above the median.

### Analysis

All analyses were run using IBM SPSS version 25.01. Since our outcome variable was dichotomous, we used the complex samples logistics regression (CSLOGISTIC) procedure. This procedure performs the analysis on the sample using complex sampling methods and weights (described above). The procedure estimates the variances based on the sampling design and reduces the number of agencies/providers back to the original sample size, thus avoiding “artificially” decreasing the standard errors and inflating the power of these analyses. In the first set of analyses, we ran logistic regression analyses for each of the independent variables, where it was the only predictor in the model. The variables with a *p*-value < .10 (two-tailed tests) were included simultaneously in the second analysis, which was a final multivariate logistic model.

## Results

For the combined sample, 33% of agencies/providers provided SUP programming to justice-involved youth. Agency/provider characteristics are presented in Table 1. BH providers were significantly more likely to provide SUP than CS agencies (45% vs. 17%; χ^2^ (1) = 41.0, *p* < .001).

**Table 1:** Agency characteristics

	% or Mean (SD)
Agency type	
Juvenile justice	43.1%
Behavioral health	56.9%
Agency location	
Urban county	36.7%
Rural county	63.3%
Number of doctoral-level full-time equivalent staff	0.3 (0.7)
Number of master’s-level full-time equivalent staff	4.1 (13.2)
Distribution of female youth in the agency’s caseload	
Females as < 30% of youth in the caseload	37.5%
Females as > = 30% of youth in the caseload	40.0%
Female distribution unknown	22.5%
Distribution of minority youth in the agency’s caseload	
Minorities as < 8% of youth in the caseload	38.6%
Minorities as > = 8% of youth in the caseload	38.0%
Minority distribution unknown	23.4%
Distribution by age in the agency’s caseload	
Youth aged 14 and older as < 86% of youth	41.4%
Youth aged 14 and older as > 86% of youth	37.4%
Age distribution unknown	21.2%
Maximum rate of use of alcohol, marijuana, or non-medical prescription drugs	
Maximum use below the median	13.0%
Maximum use above the median	47.9%
Rate of use unknown	39.1%

Note: Data are weighted to reflect the estimated national population estimate of the 3202 CS agencies plus 4252 BH service providers and in the U.S. and have been adjusted for survey non-response at the state level

A series of univariate logistic regression models of provision of SUP were performed (see Table 2, Column 1). In addition to the difference between BH providers and JJ agencies, two other agency characteristics were significantly associated with the likelihood of providing SUP programming. There was a positive association between the number of master’s level FTEs working in the agency/provider and the odds of offering SUP. While there were not significant differences by the demographic composition of the agency’s caseload, there were significant differences in the likelihood of providing SUP by the substance use characteristics of the agency’s caseload. Compared to agencies where the maximum substance use distribution was above the median, agencies/providers below the median were significantly less likely to provide SUP to youth. In addition, agencies/providers that were unable to report on the substance use distribution were significantly less likely than agencies/providers reporting maximum substance use above the median to provide SUP.

**Table 2:** Logistic regression models of provision of SUP by agency characteristics

	Univariate Models OR (95% CI)	*p*	Multivariate Models OR (95% CI)	*p*
Agency type				
Juvenile justice	**0.25 (0.10, 0.67)**	**.006**	**0.33 (0.11, 0.94)**	**.037**
Behavioral health	Reference		Reference	
Agency location				
Urban county	Reference			
Rural county	1.14 (0.49, 2.64)	.759		
Number of doctoral-level full-time equivalent staff	1.35 (0.85, 2.15)	.201		
Number of master’s-level full-time equivalent staff	**1.06 (1.01, 1.11)**	**.027**	1.05 (1.00, 1.10)	*.066*
Distribution of female youth in the agency’s caseload				
Females as < 30% of youth in the caseload	1.00 (0.33, 3.03)	.995		
Females as > = 30% of youth in the caseload	Reference			
Female distribution unknown	0.68 (0.22, 2.12)	.503		
Distribution of minority youth in the agency’s caseload				
Minorities as < 8% of youth in the caseload	0.95 (0.31, 2.94)	.935		
Minorities as > = 8% of youth in the caseload	Reference			
Minority distribution unknown	0.71 (0.27, 1.85)	.447		
Distribution by age in the agency’s caseload				
Youth aged 14 and older as < 86% of youth	1.51 (0.54, 4.25)	.430		
Youth aged 14 and older as > 86% of youth	Reference			
Age distribution unknown	0.97 (0.33, 2.82)	.948		
Maximum rate of use of alcohol, marijuana, or non-medical prescription drugs				
Maximum use below the median	**0.18 (0.04, 0.67)**	**.011**	**0.22 (0.07, 0.67)**	**.008**
Maximum use above the median	Reference		Reference	
Rate of use unknown	**0.39 (0.16, 0.97)**	**.042**	**0.33 (0.12, 0.88)**	**.027**

Notes: OR = Odds Ratio, CI = Confidence Interval, Bold indicates *p* < .05; Italics indicates *p* < .10 (two-tailed test). Only variables from the univariate analyses with a *p* < .10 were included in the multivariate model

We entered variables with *p* < .10 from the univariate analyses into a multivariate logistic regression model. (Table 2, Column 2). Controlling for master’s level staff and the substance use distribution of the caseload, CS agencies were about 67% less likely to offer SUP when compared to BH providers. However, the number of master’s level FTEs was no longer significantly associated with the odds of providing SUP once agency type and the substance use distribution were controlled. The differences for the measures of the substance use distribution remained significant in the multivariate model. Agencies/providers below the median on substance use distribution were about 78% less likely than agencies/providers above the median to provide SUP. Furthermore, agencies that were unable to provide data on the substance use measures were 67% less likely to provide SUP than agencies above the median.

## Discussion

Providing SUP in adolescence is critical to avoid worsening substance use trajectories into later adolescent and adulthood. This is particularly true of justice-involved populations for whom outcomes associated with substance use include deeper and sometimes intractable justice involvement. Yet despite the development of evidence-based SU prevention programming, we found that only a third of community supervision and behavioral health agencies provided these programs, evidence-based or otherwise. This finding is consistent with the finding of the lack of evidence-based practices in an earlier juvenile justice survey (Young, Dembo, & Henderson, 2007).

As would be expected, provision of SUP programming was driven by agency or provider reported of substance use among youth: the greater the reported use and thus service need of youth on an agency or provider’s caseload, the greater likelihood of providing preventions programming. These findings are consistent with those of Sales et al. (2018) who found that CS staff largely perceived SUP as a key part of their agency’s mission and that greater perceived importance of SUP was associated with greater likelihood of offering programs with these agencies. Nonetheless, data from this national sample found low levels of uptake of SUP among agencies serving justice-involved youth under CS.

The results also indicated that agencies/providers who could not provide youth substance use characteristics were two-thirds less likely to provide SUP. This suggests that perhaps agencies/providers should focus efforts on access and use of data to inform their needs and practices. This paper provided findings that deserve additional comment or were unexpected. First, at a descriptive level, what can be made of the fact that about one third of CS agencies and BH providers report offering substance use services to justice-involved youth? Without longitudinal data on trends in prevention availability, it is difficult to tell if this estimate should be celebrated as a glass that is one-third full or lamented given that two out of every three agencies do not provide such services. Going forward, trend data would be useful to detect gains or losses on this crucial data point.

Second, it was rather surprising to find no discernible rural-urban differences in SUP aimed at justice-involved youth. Given a wealth of previous studies that reveal pronounced health disparities, justice infrastructure gaps, and a lack of evidence-based programs in rural areas (Bartkowski et al., 2018; Saunders et al., 2016), we presumed that such differences would surface, but they were not observed. It would seem that, at least where this dimension of youth service provision is concerned, rural communities are on par with their urban counterparts. However, our measure of SUP was generated based on a list of evidence-based programs or programs that were locally developed to address needs. Additional research should be conducted to determine if there are rural-urban differences in opting for established programs that have a solid base of evidence (e.g., tested with a control group), programs that are categorized as a promising practice (i.e., some evidence typically without a control group), and those that represent a “grow your own” locally developed program that may have been more minimally evaluated. Our measure does not enable us to draw such fine-grained distinctions, but they could be important. It is possible that measures that are more refined would permit the detection of rural-urban differences. One important potential implication of this study concerns a strategic approach to youth SUP. As noted above, justice-involved youth exhibit a much higher likelihood of substance use than their peers who are not involved in the justice system. The findings reported here do suggest that evidence-based prevention services should likely be expanded beyond the roughly one in three CS agencies or BH providers that offer such services to justice-involved youth. Given the propensity for youth to recidivate at some point after their initial infraction, it seems worthwhile to make SUP services part of CS programs even when justice-involved youth do not present with a substance use disorder or a pronounced treatment need. At the very least, the effects of programmatic expansion efforts in circumscribed geographical areas could be tested against the treatment as usual modality that is driven by a disorder-based need. The relatively inexpensive cost of prevention when compared with the considerable cost of treatment could recommend the expansion of prevention services if they prove to be useful in comparisons against current approaches. An effort to expand SUP services to many or even all justice-involved youth would align well with related efforts to scale up evidence-based programs to deliver more efficacious results in a manner that proves, in the long run, to be much more cost effective (Gottfredson et al., 2018). Here again, however, scaling up efforts should be conducted with attention to local community dynamics (e.g., rural-urban locales, BH providers internal or external to CS agencies).

Several study limitations should be noted. First, the models were estimated using cross-sectional data, so causality cannot be established. A follow-up survey with these agencies is currently being fielded, so future analyses will be able to examine longitudinal data. As noted previously, trends are vital to understanding the trajectory of substance use service offerings provided to justice-involved youth. Second, all data were self-reported, which can lead to bias in over-reporting or under-reporting of agency characteristics. Third, the survey content for the juvenile justice and BH agencies were tailored to address key variables of these distinct systems, resulting in a limited number of agency characteristics that were measured for both types of agencies. It is likely that there are additional relevant unmeasured agency characteristics that may be correlated with the provision of SUP, such as funding for SUP through contracts or in educational systems. There also were substantial missing data for the caseload characteristics, where respondents were unable to provide the requested information. We considered imputation strategies, but ultimately concluded that the presence and prevalence of missing data are actually important in their own right, as they may identify agencies with less well-developed data systems, which may be a barrier to offering innovative services to meet the needs of youth.

## Conclusions

This study represents an important initial step toward providing a national snapshot of SUP services offered to justice-involved youth. In future efforts, we will continue to track this important facet of service provision to adolescent offenders to determine any marked increases, decreases, or plateaus in such offerings. Until such research can be conducted, we have established an important baseline against which trends can be assessed and upon which strategic decisions can be made to address a need that is alarmingly prevalent among justice-involved youth. A second wave of national surveys have been completed and we hope to use this data, not only to see if we can replicate this paper’s findings, but to also look at any changes in the providing of SUP by CS and BH agencies serving youth on community supervision.

## Data Availability

The datasets used and/or analyzed during the current study are available from the corresponding author on reasonable request.

## Abbreviations

BH: Behavioral health
CI: Confidence interval
CS: Community supervision
CSLOGISTIC: Complex samples logistics regression
FTE: Full-time equivalent
JJ: Juvenile justice
JJS: Juvenile justice system
OR: Odds ratio
SD: Standard deviation
SUP: Substance use prevention
